# Immobilized DNA aptamers used as potent attractors for vascular endothelial cell: *in vitro* study of female rat

**DOI:** 10.1042/BSR20182444

**Published:** 2020-01-29

**Authors:** Jung-Joon Cha, Hoyeon Lee, Miyoung Kim, Juyoung Kang, Hanlim Song, Min-Gon Kim, Jong-Hyun Lee

**Affiliations:** 1 Division of Cardiology, Department of Internal Medicine, Severance Hospital, Yonsei University College of Medicine, Seoul 03722, Republic of Korea; 2 Department of Chemistry, School of Physics and Chemistry, Gwangju Institute of Science and Technology (GIST), Gwangju 61005, Republic of Korea; 3 School of Mechanical Engineering, Gwangju Institute of Science and Technology (GIST), Gwangju 61005, Republic of Korea; 4 Department of Biomedical Science and Engineering, Gwangju Institute of Science and Technology (GIST), Gwangju 61005, Republic of Korea

**Keywords:** aptamer, ligand, SELEX, vascular endothelial cell

## Abstract

Vascular endothelial cells are essential to vascular function and maintenance. Dysfunction of these cells can lead to the development of cardiovascular disease or contribute to tumorigenesis. As such, the therapeutic modulation and monitoring of vascular endothelial cells are of significant clinical interest, and several endothelial-specific ligands have been developed for drug delivery and the monitoring of endothelial function. However, the application of these ligands has been limited by their high cost and tendency to induce immune responses, highlighting a need for alternate methods of targeting vascular endothelial cells. In the present study, we explore the therapeutic potential of DNA aptamers. Using cell-SELEX technology, we identified two aptamers with specific binding affinity for vascular endothelial cells and propose that these molecules show potential for use as new ligands for drug and biomarker research concerning vascular endothelial cells.

## Introduction

The vascular endothelium comprises the inner lining of the arterial wall and is integral to both vascular function and homeostasis [1–4]. Under normal conditions, the vascular endothelium regulates anti-thrombotic surface maintenance and hemostasis, including inhibition of thrombus cascade, both synthesis and inhibition of platelet aggregation, promotion of fibrinolysis, and maintenance of the intravascular blood flow through the production of nitric oxide synthase [1,5].

The vascular endothelium is also intricately linked to disease processes such as neovascularization, the process by which tumor cells achieve a growth advantage and proliferative autonomy [6]. Specific ligands, including peptides and monoclonal antibodies, have been developed to enhance tumor killing through the disorder or suppression of endothelial cell proliferation [7]. Vascular endothelial growth factor, one of the key factors inducing neovascularization, has been widely investigated in anti-tumor research [8,9]. In addition, the anti-cancer effect can be achieved by increasing the concentration of the drug acting on the cancer cells by increasing the concentration of the anti-cancer drug around the neovascular endothelial cells [6].

Vascular endothelial dysfunction is also known to be a major cause of atherosclerosis and cerebral infarction [10]. Free radicals, diabetes, tobacco exposure, bacterial infection, oxidized low-density lipoproteins, and interventional treatments such as angioplasty or stenting can induce chronic endothelial injury [11]. This injury attracts macrophages to the arterial lining and promotes the adhesion of circulating inflammatory cells, predominantly monocyte-derived macrophages, activated T cells, and mast cells [1–3]. In an approach similar to that for tumor treatment, a specific ligand targeting tumor necrosis factor-α was designed and presented as a targeting ligand for drug delivery and imaging of atherosclerosis [12].

However, while these existing ligands do offer opportunities for specific drug delivery and treatment, they remain limited by their high cost of production, stimulation of undesirable immune responses, and irreversible denaturation [13]. A solution to these obstacles may lie in the use of aptamers, which have been introduced as promising tools for a variety of biomedical applications including diagnosis, therapy, and biomarker discovery [14,15]. Aptamers are short, single-chain nucleic acids that fold into defined 3D structures and bind a variety of ligands. They are identified by a repetitive enrichment process called SELEX, in which ligands are exponentially enriched by interacting with homologous target molecules with high affinity and specificity [16]. Aptamers bind to a large variety of target structures such as proteins, small molecules, peptides, or whole cells. Over the past decade, aptamers have been developed to meet the demand for the creation of molecular targeting tools aimed at specific cells and cell types [15].

In the present study, we use the cell-SELEX approach to identify DNA aptamers that specifically target normal vascular endothelial cells. We anticipate that these molecules will significantly enhance the development of tools including targeted drug therapies and new imaging modalities.

## Materials and methods

### Reagents

Female rat aortic endothelial cells (RAOEC), rat endothelial cell growth media, and subculture reagents [Hanks’ balanced salt solution (HBSS), trypsin-EDTA, and trypsin neutralizer] were purchased from Cell Applications, Inc. (San Diego, CA). All DNA sequences used in the present study were synthesized by Integrated DNA Technologies, Inc. (Coralville, IA). Vybrant DiO Cell-Labeling Solution was purchased from Life Technologies (Foster City, CA). Streptavidin-horseradish peroxidase (STA-HRP) and 3,3′,5,5′-tetramethylbenzidine (TMB) substrate reagent sets were obtained from BD Bio Sciences (San Diego, CA).

### Cell culture

RAOECs were maintained in rat endothelial cell growth media in a humidified incubator with 5% CO_2_ at 37°C. Cells were fed every 2–3 days. The cells were subcultured once they reached 80% confluence. For cell-SELEX, 5 × 10^4^ cells in 3 ml of rat endothelial cell growth media were seeded in a 60 cm^2^ cell culture dish (Corning) and grown for 4 days to be confluence. For confocal microscopy imaging, cells were seeded in a glass-bottom confocal dish (SPL) at a concentration of 5 × 10^4^ cells per dish and grown for 24 h. Before each experiment, cells were washed three times using HBSS.

### ssDNA library and primers

An ssDNA library consisting of sequences 40 or 50 nucleotides in length was produced using the forward primer 5′-ATA CCA GCT TAT TCA ATT-3′ (18 nt, 28% GC, *T*_m_: 45°C) and reverse primer 5′-[PHOS]AGA TTG CAC TTA CTA TCT-3′ (18 nt, 33% GC, *T*_m_: 47°C) in polymerase chain reaction (PCR) for cell-SELEX. After PCR, dsDNA was denatured to ssDNA at 95°C for 5 min, and then stored at ice for 15 min before used. The selection rounds were monitored by band thickness following DNA electrophoresis.

### SELEX and aptamer synthesis

**Figure 1:**
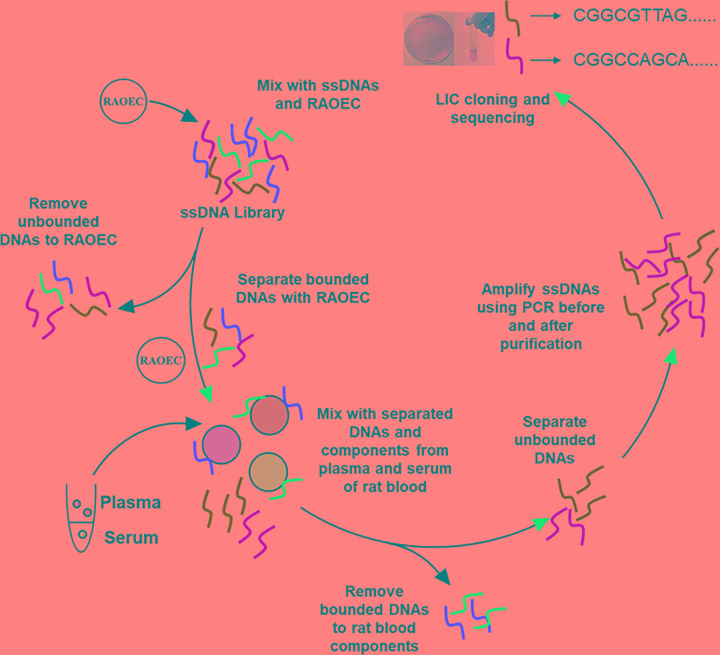
Schematic representation of cell-SELEX for aptamer selection

SELEX selection was conducted as previously described [15] employing modifications as follows to optimize the target cell type. A schematic of the aptamer selection process is shown in Figure 1. The ssDNA pool (0.25 mM, 20 µl) was diluted into 350 µl HBSS buffer before being denatured at 95°C for 5 min, cooled on ice for 20 min, and then incubated with 2 × 10^7^ RAOECs suspended in HBSS on ice using a rotary shaker for 1 h. After centrifugation at 150×***g*** for 3 min at 4°C, unbound ssDNA was removed by washing with HBSS. The bound ssDNAs were dissociated from the cell pellet and eluted by heating at 95°C for 5 min in binding buffer followed by cooling on ice for 20 min before centrifugation at 13100×***g*** for 5 min. The negative selection was performed by incubating the supernatant containing the selected ssDNAs with plasma from rat blood at room temperature for 1 h, followed by centrifugation at 13100×***g*** for 5 min at 4°C to remove bound DNAs. DNAs remaining in the supernatant were amplified by PCR twice using forward and reverse primers (Table 1) to obtain aptamers with highly selective binding specificity for ROAEC. For using rat blood, animal experiments were performed at Neuro Modulation Laboratory in Gwangju Institute of Science and Technology (GIST) (Gwangju, Republic of Korea) according to GIST institutional guidelines, and all procedures for this research were approved by the Institutional Animal Care and Use Committee at GIST (GIST-2017-015). All surgeries were performed under inhalation anesthesia with isoflurane gas or intramuscular injection of ketamine hydrochloride and xylazine, and all efforts were made to minimize suffering. ARRIVE guidelines were followed in the preparation of the manuscript.

**Table 1:** Reagents and volume of the reaction mixture of sequential PCR

Reagents	Reaction mixture (µl)
**(A) First PCR condition**	
5× PCR buffer	74
dNTP mixture (2 mM each)	19 (0.1 mM)
Primer (F, R)	18 (0.5 µM)
DNA selected pool (template)	185
DNase free water	73
Taq polymerase	1
**(B) Second PCR condition**	
5× PCR buffer	5
dNTP mixture (2 mM each)	4
Primer (F, R)	2.5
DNA pool (Amplicon)	5
DNase free water	33.35
Taq polymerase	0.15

Aptamers were purified by DNA electrophoresis followed by PCR purification (Table 2) before being cloned into the pET His6 TEV LIC cloning vector (Addgene, Cambridge, MA, U.S.A.) and transformed into *Escherichia coli* DH5α cells. Amplified plasmid DNA was isolated and then sequenced by Macrogen Inc. (Seoul, Korea). Fluorescence-labeled aptamers were purchased from Integrated DNA Technologies, Inc. (Coralville, IA) as a lyophilized powder and resuspended in HBSS at a final concentration of 100 µM. Until doing an experiment, all the aptamers resuspended HBSS at a concentration of 100 µM are stored at −20°C. Just before confocal imaging experiments, they were diluted in HBSS to a final concentration of 5 µM. A control aptamer of the same length but a randomized scrambled DNA sequence was also synthesized from Integrated DNA Technologies, Inc (Coralville, IA).

**Table 2:** Reagents and volume of the maker, eluted ssDNA, and negative control of sequential PCR

Reagents	Marker (µl)	Eluted ssDNA (µl)	Negative control (µl)
**DNA electrophoresis**			
Gel star loading dye (6×)	1	1	1
PCR product	0	5	0
PCR negative control (cell lysate)	0	0	5
Ladder (50 bp)	1	0	0
DNase free water	4	0	0

### Measurement of cell viability

Cell viability assay was performed based on measurement of the absorption of formazan crystals from 3-(4,5-dimethyltiazol-2-yl)-2,5-diphenyltetrazolium bromide (MTT), formed by intracellular oxidoreductases in the cells. RAOECs were seeded at 2 × 10^4^ cells/well in 96-well plates overnight in the cell incubator. The cells treated with various concentrations of VE aptamer 1 or VE aptamer 2 were incubated for 24, 48, and 72 h in a humidified atmosphere containing 5% (v/v) CO_2_ at 37°C. After treatment with 10 μl MTT solution (0.5 mg/ml), the final concentration was added to each well and incubated for 2 h at 37°C to allow the formation of formazan crystals. Subsequently, the remaining solution was removed to leave the cells behind in the wells, and 100 μl dimethyl sulfoxide (DMSO) was added to each well to dissolve the crystals. The absorbance of the dissolved crystals from intact cells was measured at 570 nm using a microplate reader from Cytation 5.0 Plate Reader (BioTek, Winooski, VT). To calculate the percentage of dead cells, negative control of 100% live cells was used. Cells in the control wells were not treated and cultured using cell culture media with 10% FBS before addition of MTT.

$$\text{cell}\;\text{viability}\;(\%)=\frac{\text{the}\;\text{absorbance}\;\text{of}\;\text{the}\;\text{experimental}\;\text{group}}{\text{the}\;\text{absorbance}\;\text{of}\;\text{the}\;\text{negative}\;\text{control}\;\text{group}}\times \;100$$

The average of the absorbance values for the negative control was set as a blank value.

### Confocal microscopy

For membrane staining, Vybrant DiO Cell-Labeling Solution was applied to the cells at a dilution of 1:100 in growth media for 10 min at 37°C in the cell culture incubator. After washing twice with 37°C growth media for 5 min, cells were incubated with either 100 µl of Cy5-labeled aptamers (5 µM), or 100 µl Cy5-labeled scrambled DNA control (5 µM) in HBSS for 5 min at 37°C. Cells were washed twice with HBSS for 5 min before being mounted with Dako Fluorescence Mounting Solution (Agilent Dako, Santa Clara, CA, U.S.A.).

Vybrant Cell-Labeling Solutions and Cy5 were excited at 488 nm argon-ion laser and a 633 helium–neon laser, respectively. Images were taken on an Olympus FV1000 confocal laser scanning microscope (Shinjuku-ku, Tokyo, Japan) using both 40× NA 1.00 and 60× NA 1.40 oil immersion objectives. To examine aptamer binding, 3–5 regions were imaged from each sample dish. The data were obtained, processed, and analyzed using FLUOVIEW software (Olympus). Fluorescence intensity was determined from five different images taken using the 40× objective and measured using ImageJ software (U. S. National Institutes of Health, Bethesda, Maryland, U.S.A.).

### Direct enzyme-linked oligonucleotide assay (ELONA)

RAOECs (10^4^ cells/well) were loaded onto 96-well plates with cell media in a 37°C cell incubator for overnight. After washing twice with HBSS to remove the cell media, cells were treated with biotin-labeled VE aptamer 1 and VE aptamer 2 (0, 0.128, 0.64, 3.2, 16, 80, and 400 nM) with a binding buffer for 5 min at 37°C. Each well was then washed three times with 200 μl HBSS and STA-HRP (1 μg/ml) was added to the wells and allowed to react at 37°C for 30 min, followed by reaction with TMB solution for 20 min at 37°C for signal detection. The absorbance of each well was measured at 650 nm. The binding affinities between VE aptamer 1 or VE aptamer 2 and RAOECs were calculated by dissociation constant (*K*_d_) based on absorbance values at 650 nm using GraphPad Prism version 6 software for Windows (GraphPad Software, La Jolla, CA, www.graphpad.com). *K*_d_ values of increasing concentrations of aptamer (VE aptamer 1 or VE aptamer 2) were plotted as the abscissa using a ‘saturation one-site’ fitting model.

## Results and discussion

We sequenced several aptamer candidates, two of which (VE aptamer 1 and VE aptamer 2) by cell-SELEX (Figure 1) were sufficiently reproduced for further sequence analysis and binding studies. The full-length VE aptamer 1 and VE aptamer 2 sequences were determined to be 5′-CGGCGCGCAG AGTGGAGTCG GACGGAGCTG TTTGGTGGTG T-3′, and 5′-CGGCCAGCAC AGAGAAAGTA CAGCGGCCCA CTCATCGGCG A-3′, respectively.

**Figure 2:**
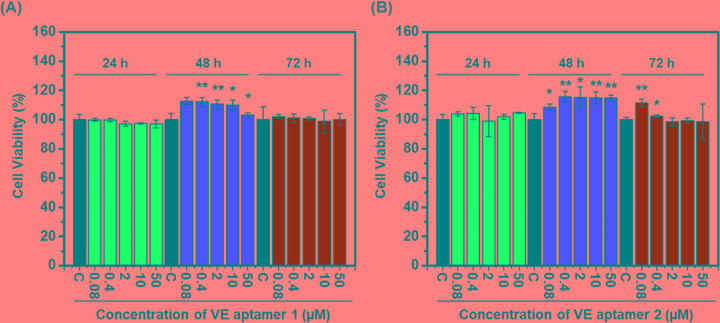
Effects of VE aptamer 1 or VE aptamer 2 on cell viability in RAOECs The viability of cells pre-treated with (**A**) VE aptamer 1 or (**B**) VE aptamer 2 and that were incubated for 24, 48, or 72 h depending on different concentrations. Dark bars represent the control that was only incubated with media. After treatment of the samples during each of the incubation time points, the visible absorption of formazan crystals was measured at 570 nm. The cell viabilities were normalized by the control value ([VE aptamer 1 or VE aptamer 2] = 0, control). Results represent the mean [±standard error of mean (SEM)] of three independent experiments (*n*=3). The statistical signification was marked as * and ** for *P*<0.05 and *P*<0.01, respectively, compared with the control.

To investigate the toxicity of VE aptamer 1 (Figure 2A) and VE aptamer 2 (Figure 2B) to RAOECs, the cell viability test was conducted in various conditions, which up to 50 µM VE aptamer 1 and VE aptamer 2 were incubated with the RAOECs for 72 h. Even in the harshest conditions tested, both VE aptamer 1 and VE aptamer 2 showed approximately 100% survival when compared with the control group, which treated with only cell media without aptamers. In this test environment, more aptamer was reacted with target cells for a longer time than the conditions for binding the aptamer and the cell. Thus, VE aptamer 1 (5 µM) and VE aptamer 2 (5 µM) can use as a potent attractor with very low toxicity in the environment in which cells live.

**Figure 3:**
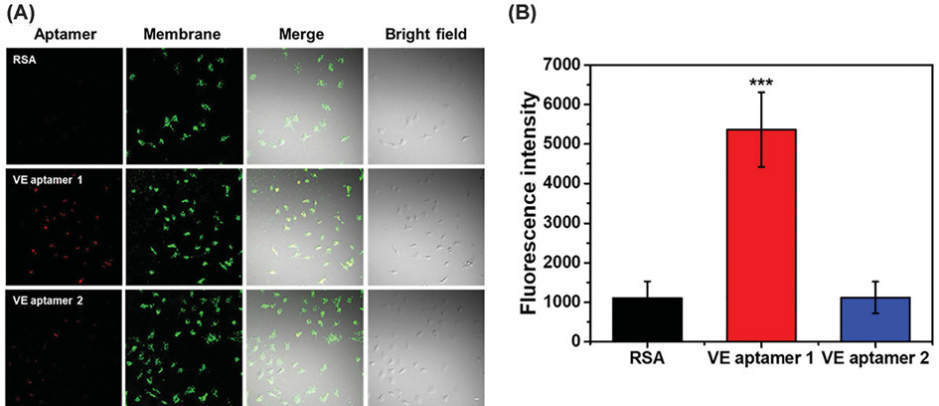
Binding test of selected aptamers with RAOECs (**A**) Confocal microscope images with VE aptamer 1 (5 µM) and VE aptamer 2 (5 µM) conjugated with Cy5 to use to live-stain RAOEC. A randomized scramble derivative of the aptamer RSA (5 µM) conjugated with Cy5 was used to stain RAOEC as a control group. After treated the Cy5 labeled VE aptamer 1, VE aptamer 2, and RSA washed twice with HBSS buffer, all fluorophores were excited at 488 nm argon-ion laser and a 633 helium–neon laser, respectively. Images were taken on an Olympus FV1000 confocal laser scanning microscope using both 40× NA 1.00 and 60× NA 1.40 oil immersion objectives. Scale bar represents 10 µm. (**B**) Fluorescence intensities of Cy5 labeled VE aptamer 1, VE aptamer 2, and RSA were measured using ImageJ software based on the confocal microscope images. Results represent the mean [±standard error of mean (SEM)] of three independent experiments (*n*=5). The statistical signification was marked as *** for *P*<0.001, compared with the control.

To determine whether the selected aptamers bind to RAOECs, after washing aptamers that were not bound to the RAOECs, we checked the fluorescence intensities with cyanine 5 (Cy5) conjugated VE aptamer 1 and VE aptamer 2 that only were attached the RAOECs using confocal microscopy. A randomized scramble derivative of the aptamer (RSA), which had a randomized sequence of equivalent length and labeled to the same fluorophore staining for RAOECs, was used to exclude nonspecific aptamer binding. Vybrant DiO Cell-Labeling probe was used to stain the cell membranes. The fluorescence signal of Cy5 labeled VE aptamer 1 increased significantly in RAOECs when compared with that of the Cy5 labeled RSA. Figure 3A shows the confocal images of RAOECs stained with the various fluorescence-labeled aptamers. Figure 3B displays the average fluorescence intensity of 15 cells imaged with confocal microscopy from five different spots in each RAOECs cultured dish. Fluorescence intensity of VE aptamer 1 was significantly higher, at a 5-fold increase than that of the RSA. However, there was no significant difference in fluorescence intensity between VE aptamer 2 and the RSA. Additionally, the yellow color in the merge image that appeared as the overlapped fluorophore signals of the VE aptamer 1 and Vybrant DiO Cell-Labeling probe indicated that the VE aptamer 1 was located on the RAOECs membrane (Figure 3A). Therefore, VE aptamer 1 adhered to the cell membrane of the RAOECs. On the other hand, VE aptamer 2 could be inferred that its binding strength was weak, although it binded to the RAOECs the same as VE aptamer 1.

**Figure 4:**
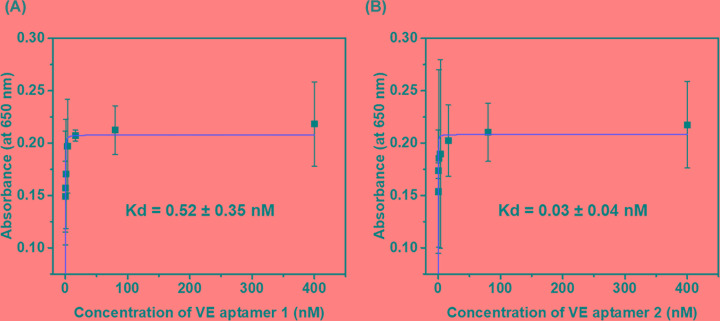
The binding affinity of aptamer candidates using ELONA RAOECs (10^4^ cells/well) were treated with various concentration of biotinylated (**A**) VE aptamer 1 or (**B**) VE aptamer 2. STA-HRP (1 μg/ml) was added to the cells, followed by reaction with TMB solution signal detection (absorbance was measured at 650 nm). Results represent the mean (±SEM) of three independent experiments (*n*=3). Red lines in the graphs represent the nonlinear fitting curve with Michaelis–Menten enzyme kinetic model function.

To know the binding affinity if the VE aptamer 1 and VE aptamer 2, direct ELONA (Figure 4) was performed under the various concentration of VE aptamer 1 and VE aptamer 2 (0, 0.128, 0.64, 3.2, 16, 80, and 400 nM) with HBSS binding buffer. The binding curves were obtained by measuring absorbance wavelength at 650 nm of TMB which reacted with STA-HRP associated with biotinylated VE aptamer 1 and VE aptamer 2 bound ROAECs. The dissociation constants (*K*_d_) were calculated by a fitting curve that is one site-specific binding model based on Michaelis–Menten equation. *K*_d_ of VE aptamer 1 (0.52 ± 0.35 nM) exhibited higher than VE aptamer 2 (0.03 ± 0.04 nM).

The results of the present study provide the first report of aptamer, which had a specific binding affinity to RAOECs. Furthermore, the found aptamers are anticipated to facilitate the development of new targeted drug therapies, biomarker discovery, and imaging of coronary, cerebral, and peripheral artery angiograms. Although MacLean et al. had reported that human-derived cyclic peptide has a similar effect on vasoconstriction of rat [17], whether the aptamers have specific affinity in human endothelial cells is still uncertain due to interspecies difference in the various cells of rat and of human [18]. Therefore, future investigation of the interaction between the aptamers and human vascular endothelial cells may clarify the potential of the aptamers. Additionally, *in vivo* studies, or *in vitro* analyses employing tissue samples of rat vascular endothelium, specificity against smooth muscle cells, and isolation and efficacy related to the cell from males, will further confirm the *in vivo* potential of these aptamers to recognize rat vascular endothelial cells.

## Abbreviations

ELONA: enzyme-linked oligonucleotide assay
HBSS: Hanks’ balanced salt solution
PCR: polymerase chain reaction
RAOEC: rat aortic endothelial cell
RSA: randomized scramble derivative of the aptamer
STA-HRP: streptavidin-horseradish peroxidase
TMB: 3,3′,5,5′-tetramethylbenzidine
